# Possible Involvement of Hsp90 in the Regulation of Telomere Length and Telomerase Activity During the *Leishmania amazonensis* Developmental Cycle and Population Proliferation

**DOI:** 10.3389/fcell.2021.713415

**Published:** 2021-10-28

**Authors:** Beatriz C. D. de Oliveira, Mark E. Shiburah, Stephany C. Paiva, Marina R. Vieira, Edna Gicela O. Morea, Marcelo Santos da Silva, Cristiane de Santis Alves, Marcela Segatto, Fernanda Gutierrez-Rodrigues, Júlio C. Borges, Rodrigo T. Calado, Maria Isabel N. Cano

**Affiliations:** ^1^Department of Chemical and Biological Sciences, Institute of Biosciences, São Paulo State University (UNESP), São Paulo, Brazil; ^2^Faculdade Brazileira Multivix, Vitória, Brazil; ^3^Hemocentro da Faculdade de Medicina de Ribeirão Preto, Universidade of São Paulo, São Paulo, Brazil; ^4^São Carlos Institute of Chemistry, University of São Paulo, São Paulo, Brazil

**Keywords:** *Leishmania* life forms, continuous *in vitro* passages, telomeres maintenance, telomerase ribonucleoprotein complex, LHsp90

## Abstract

The *Leishmania* developmental cycle comprises three main life forms in two hosts, indicating that the parasite is continually challenged due to drastic environmental changes. The disruption of this cycle is critical for discovering new therapies to eradicate leishmaniasis, a neglected disease that affects millions worldwide. Telomeres, the physical ends of chromosomes, maintain genome stability and cell proliferation and are potential antiparasitic drug targets. Therefore, understanding how telomere length is regulated during parasite development is vital. Here, we show that telomeres form clusters spread in the nucleoplasm of the three parasite life forms. We also observed that amastigotes telomeres are shorter than metacyclic and procyclic promastigotes and that in parasites with continuous *in vitro* passages, telomere length increases over time. These observed differences in telomere length among parasite’s life stages were not due to lack/inhibition of telomerase since enzyme activity was detected in all parasite life stages, although the catalysis was temperature-dependent. These data led us to test if, similar to other eukaryotes, parasite telomere length maintenance could be regulated by Hsp83, the ortholog of Hsp90 in trypanosomatids, and *Leishmania* (LHsp90). Parasites were then treated with the Hsp90 inhibitor 17AAG. The results showed that 17AAG disturbed parasite growth, induced accumulation into G2/M phases, and telomere shortening in a time-dependent manner. It has also inhibited procyclic promastigote’s telomerase activity. Besides, LHsp90 interacts with the telomerase TERT component as shown by immunoprecipitation, strongly suggesting a new role for LHsp90 as a parasite telomerase component involved in controlling telomere length maintenance and parasite life span.

## Introduction

Leishmaniases are a group of infectious diseases caused by parasites of the genus *Leishmania*, affecting millions of people worldwide. The disease has a broad spectrum of clinical manifestations varying from asymptomatic, self-limited skin lesions to systemic and fatal infections (Kevric et al., 2015). The parasite’s sequential developmental cycle involves three main life forms: procyclic promastigotes (PP), metacyclic promastigotes (MP), and amastigotes (Am). In general, the non-infective (but highly proliferative) PP lives in the phlebotomine sandflies’ midgut (invertebrate host). They can transform into non-proliferative MP, infecting mammals (vertebrate hosts) and transforming into Am. Am live and multiply inside the vertebrate host cells and infect new cells and other sandflies in new rounds of infection (Sacks and Perkins, 1984; Alvar et al., 2012). Therefore, to survive and complete the developmental cycle, *Leishmania* spp. needs to adapt to drastic environmental changes during the transition from invertebrate to the vertebrate host. Alterations in the temperature and pH, which affect cell morphology, cell surface, and metabolism, including gene expression and the activity of some enzymes, are among the challenges parasites pass through to survive (Sacks and Kamhawi, 2001; Wheeler et al., 2010; Dillon et al., 2015; Inbar et al., 2017). Interestingly, Am and MP, the infective forms, are close-related regarding gene expression than PP, which cannot infect cells (Inbar et al., 2017).

Despite its global distribution, to date, there are few strategies to treat and control leishmaniasis. Most antileishmanial treatments are based on drugs developed several years ago, presenting side effects and leading to drug resistance. Also, few human vaccines in clinical trials demonstrate the urgency of finding new anti-parasite therapies that are less toxic and more active (Hotez et al., 2016; Moafi et al., 2019; World Health Organization [WHO], 2020). Thus, an increasing interest in studying telomeres as drug design targets has emerged (Olaussen et al., 2006; Chen et al., 2009; Sekaran et al., 2014; Dey and Chakrabarti, 2018). Telomeres are protein-DNA complexes that protect chromosome ends from degradation and fusion (Giardini et al., 2014). Chromosome ends in *Leishmania*, like most eukaryotes, are composed of conserved 5’-TTAGGG-3’ telomeric repeats maintained by telomerase (Fu and Barker, 1998; Cano et al., 1999; Conte and Cano, 2005). Telomerase is a ribonucleoprotein enzyme minimally composed of two catalytically subunits: the telomerase reverse transcriptase protein (TERT) and the telomerase RNA (TER), which contains the template that specifies the sequence of the telomeric repeats (Feng et al., 1995; Giardini et al., 2014). Both components were already identified and partially characterized in *Leishmania* sp., showing conserved and genus-specific features (Giardini et al., 2006; Vasconcelos et al., 2014). For example, the *Leishmania* telomerase RNA component (LeishTER), similarly to *Trypanosoma brucei*, was shown to be processed by *trans*-splicing. Still, its primary sequence, including the template sequence, is only conserved within the *Leishmania* genus (Vasconcelos et al., 2014). Also, although the purified parasite’s telomerase activity presents catalytic properties shared with telomerase described in model organisms (Cano et al., 1999), it also contains enzymatic characteristics specific to the genus (Giardini et al., 2011). All these identified features can elect parasite telomerase as a potential drug target.

Here we show that in *L. amazonensis*, telomeres’ foci are spread in the nucleoplasm of all life stages. Intriguingly, telomeres from Am and MP are shorter than PP, and telomere length increases in parasites with continuous *in vitro* passages. However, telomerase activity was detected in all parasite life stages. Furthermore, it showed to be life-stage dependent, strongly suggesting that parasite telomere length is regulated during development by a still unknown factor. Therefore, based on the literature, we speculate if Hsp90, considered one of the players involved in controlling telomerase activity in model organisms, could be implicated in parasite telomere maintenance (Holt et al., 1999; Forsythe et al., 2001; Keppler et al., 2006; Toogun et al., 2008; Viviescas et al., 2019). The ortholog of Hsp90 in *Leishmania* spp. and other trypanosomatids is Hsp83, which is important for parasite growth and the differentiation of PP into Am forms (Brandau et al., 1995; Wiesgigl and Clos, 2001; Zilka et al., 2001). Besides, it is known that heat shock stress and the inhibition of *Leishmania* Hsp90 (LHsp90) by geldanamycin and its analogs induce parasite death and, in some species, apoptosis-like death (Wiesgigl and Clos, 2001; Li et al., 2009; de Petersen et al., 2012; Santos et al., 2014). To test the hypothesis that LHsp90 could regulate telomere length maintenance in *L. amazonensis*, we treated parasites with 17AAG, a geldanamycin analog. 17AAG inhibits most Hsp90 by binding the ATP/ADP domain located at the N-terminal region of the protein (Grenert et al., 1997). In *Leishmania* spp., 17AAG shows a stronger preference to bind the N-terminal domain of LHsp90 (Palma et al., 2019). Our findings suggest that LHsp90 plays a conserved role in *L. amazonensis* telomeres maintenance since its inhibition impaired parasite growth, induced cell cycle arrest, caused telomere shortening, and inhibited *in vitro* telomerase activity. Besides, LHsp90 co-immunoprecipitated with the telomerase TERT component, strongly suggesting that it is part of the complex and may be involved in parasite telomere length maintenance.

## Methods

### Parasites

All three *L. amazonensis* (strain MHOM/BR/1973/M2269) life forms were obtained from the same developmental cycle. PP was obtained from lesion-derived Am and cultivated at 28°C in M199 medium (Gibco) supplemented with 10% fetal calf serum (Cultilab). MP was obtained from PP stationary cultures using agglutination with peanut lectin (Sacks and Perkins, 1984). Am was obtained from mice footpad lesions as described before (Barbiéri et al., 1990). PP passage 1 (P1) was obtained from newly *in vitro*-transformed PP and was maintained in continuous passages. PP from passages 2, 4, 6, and 8 represent the continuous cultivation (every 4 days) of PP from passage 1 in exponential growth. MP from passages 1, 2, 4, 6, and 8 were obtained from PP passages 1, 2, 4, 6, and 8 in the stationary growth phase (tenth day).

### Telomere Fluorescence *in situ* Hybridization

The telomere fluorescence *in situ* hybridization was performed as described before (Siqueira Neto et al., 2007; da Silva et al., 2010), with minor modifications. Exponentially growing *L. amazonensis* PP from passage 6 (∼ 1 × 10^6^ cells), ∼ 1 × 10^5^ MP from passage 6, and ∼ 1 × 10^5^ lesion-derived Am were harvested at 2,500 x g for 5 min at 4∘C, washed in PBS (137 mM NaCl, 2.7 mM KCl, 10 mM Na_2_HPO_4_, and 2 mM KH_2_PO_4_, pH 7.4) and fixed in 1% formaldehyde for 5 min at 4°C. After permeabilization with 0.1% Triton X-100 for 10 min at room temperature, the parasites were incubated with 0.1 M glycine for 5 min at room temperature. Parasites were attached to glass coverslips, and FISH reactions were done using an 18-mer PNA (Peptide Nucleic Acid) FITC-labeled telomeric oligoprobe (CCCTAA)_3_ (PANAGENE). DNA in the nucleus and kinetoplast were stained with Vectashield^®^ mounting medium DAPI (Vector Labs). The images were analyzed using a Nikon 80i fluorescence microscope and were superimposed using NIS elements software (v. Ar 3.10).

### Estimation of 17AAG IC_50_ for *L. amazonensis* Procyclic Promastigotes

To estimate the IC_50_ (Half-maximal inhibitory concentration) of 17AAG (Cayman Chemical) for *L. amazonensis* PP (passage 6), we used PrestoBlue (Invitrogen), supplied as a 10X solution, according to the manufacturer’s instructions. Briefly, exponentially growing PP from passage 6 (∼3 × 10^6^ cells) in M199 medium supplemented with 10% FCS (80 μL), were incubated with 10 μL of increased concentrations of 17AAG (25, 50, 100, 150, 200, 250, 300, 350, 400, and 500 nM) diluted in 90% methanol and deposited in 96 well plates. After 48 h at 28°C, 10 μL of 10X PrestoBlue was added to each well followed by incubation for 10 min at 28°C. The viability of the cells was detected by measuring Absorbance 570 nm. The percentage of viable parasites was plotted against the log of the drug concentration, and the IC_50_ was analyzed using GraphPad Prism 8.0. Assays were done in triplicate, and as controls, we used wild-type cells (wt) and methanol-treated cells (meth-treated).

### Telomeric Southern Blotting Analysis

Genomic DNA (gDNA) from the three *L. amazonensis* life forms, PP and MP from continuous passages, and PP (passage 6) tested in all different conditions were obtained using the DNeasy Blood and Tissue kit (Qiagen). The integrity of each DNA sample was confirmed by fractionation in 0.8% agarose gels (Supplementary Figures 1A,B). DNA samples (1.0 μg each) were resuspended in 1X TE and digested with 10 U *Afa*I (Thermo Scientific) at 37°C overnight to liberate the chromosome end termini (Conte and Cano, 2005). Terminal restriction fragments (TRF) were fractionated onto a 0.8% agarose gel and transferred to Hybond N + nylon membranes. Southern blots were done using *Afa*I-digested DNA fragments hybridized with a DIG-labeled telomeric (TEL) probe (5′-TTAGGG_3_-3′). Membranes were stripped and re-hybridized with a PCR fragment of *L. amazonensis* GAPDH (Fw: 5′ GAAGGACTGGCGCGGTGGCCGCGCG 3′, and Rv: 5′ CCACGGCCTTGGCGGCGCCGGTCG 3′) labeled using the PCR DIG probe synthesis kit (Roche). The hybridization signals were developed with an anti-DIG-HRP conjugate antibody (Roche) and CPD-Star (Roche). The average TRF was determined by comparing the TRF location on the blot relative to the DNA molecular weight marker VII DIG-labeled (Roche).

### Flow Cytometer Analysis

Three biological replicates of each sample: *L. amazonensis* PP from passage 6 (∼ 2 × 10^6^ cells) in exponential growth (wt), meth-treated, and treated for 48 and 96 h with 100 and 200 nM 17AAG were analyzed. Parasite samples were harvested at 2,500 x g for 5 min at 4∘C, washed in PBS, fixed in 90% methanol for 30 min at −20∘C, washed again with PBS, and suspended in PBS. Cells were incubated with 10 μg/ml RNase A (Invitrogen) for 30 min at 37°C. DNA content in each cell cycle phase was estimated by staining cells with 10 μg/ml propidium iodide for 40 min at 37°C (Sigma) followed by flow cytometry analysis using Accuri C6 plus flow cytometer (BD Biosciences). Flow Jo was used for data analysis and to construct the histograms (events x *FL2 area*). A total of 20,000 events were analyzed for each sample with a similar variance between the groups. Raw data from flow cytometry experiments are available from the corresponding author on request.

### Telomeric Flow- Fluorescence *in situ* Hybridization

Flow-Fish was used to quantitatively estimate telomeres length from the three *L. amazonensis* life forms in different conditions: meth-treated PP (passage 6) and PP (passage 6) treated for 48 and 96 h with 100 and 200 nM 17AAG. Assays were done in triplicates using three biological replicates of each sample treated on different days, using a modification of the Baerlocher et al. method (Baerlocher et al., 2006; da Silva et al., 2017). As described before (da Silva et al., 2017), human leukocytes (1 × 10^6^ cells) with a known telomere length were mixed with each parasite sample to allow proper discrimination of parasites and controls. FISH reactions were done using an 18 mer PNA FITC-labeled telomeric oligoprobe (CCCTAA)3 (PANAGENE). Results were analyzed using Accuri C6 plus flow cytometer (BayBioscences, Kobe, Japan). Quantum FITC beads (Bangs Laboratories) were used to calculate the fluorescence recorded for each sample, the MFI (Mean Fluorescence Intensity), which was converted into equivalent MESF (Molecules Equivalent of Soluble Fluorochrome) according to Baerlocher et al. (2006). The results were also represented as fluorescent histograms, and the morphometric parameters (FSC-area x FSC-height) allowed to discriminate and select cells within different groups. Raw data from flow fish experiments are available from the corresponding author on request.

### Protein Extracts, Immunoprecipitation, and Western Blot Analysis

Protein extracts were obtained from *L. amazonensis* lesion-derived Am and, PP and MP both from passage 6. Cells (∼ 1 × 10^9^) were harvested by centrifugation, washed in ice-cold PBS supplemented with 2% glucose (PBS-G), and resuspended in buffer A (20 mM Tris-HCl pH 7.5, 1 mM EGTA pH 8.0, 1 mM EDTA pH 8.0, 1 mM spermidine, 0, 3 M spermine, 1 mM DTT, 15 mM NaCl) containing 1 X protease inhibitor cocktail (Sigma), followed by incubation for 30 min on ice. Protein extracts were prepared in buffer A containing 0.5% NP-40 v/v. Total protein concentration of each extract was determined by reading OD280nm. Finally, telomerase positive extracts were purified in DEAE-sepharose columns using a standard protocol (Cano et al., 1999; Giardini et al., 2011).

Western blot assays (Supplementary Figure 4) were done with approximately 300 μg of each telomerase-positive extracts fractionated in 10% SDS-PAGE and transferred to nitrocellulose membranes (BioRad). Extracts were probed with rabbit-produced polyclonal sera against recombinant *L. amazonensis* TERT (N-terminal region) (1:8,000 dilution) (produced by our research group), and recombinant *L. braziliensis* Hsp90 (1:5,000 dilution) (Seraphim et al., 2013). As secondary antibody, we used goat anti-rabbit HRP-conjugated (Bio-Rad) (1:30,000 dilution) and ECL reagent (GE).

Co-immunoprecipitation assays were performed using 20 μg of each telomerase positive protein extracts (input), 5 μg each polyclonal anti-LbHsp90 or anti-LaTERT sera, and DynaBeads protein A (Thermo Fisher). Protein extracts were incubated at 37∘C for 2 h with protein A-coupled with the specific serum in the presence of IP buffer (25 mM TRIS-HCl pH 8.0, 2 mM EDTA pH 8.0, 10% glycerol, 1 X protease inhibitor cocktail). Samples were collected and fractionated in 10% SDS-PAGE. The presence of TERT and Hsp90 in the Co-IP fractions was detected by western blot revealed with anti-LbHsp90 (1:5,000 dilution) and anti-LaTERT (1:8,000 dilution) sera.

### Telomerase Activity Measured by Telomere Repeat Amplification Protocol Assay

Telomerase activity assay was performed using a modified one-tube telomere repeat amplification protocol (TRAP) assay and DEAE semi-purified extracts (Cano et al., 1999; Giardini et al., 2011) obtained from the three life forms (Am and, PP and MP both from passage 6). A 5′-DIG-labeled TS primer (5′-AATCCGTCGAGCAGAGTT-3′) was used as the template for the telomerase activity step. The Cx-extend primer [5′-GTG (CCCTTA)_3_CCCTAA-3′] was used as the reverse primer in the PCR reaction step. The amount of protein in the DEAE semi-purified extracts used in each reaction was around 1 μg and control reactions were done without extracts or TS primer. Telomerase activity was tested by pre-incubating the extracts with 200 ng DNase-free RNase A (Sigma) for 5 min at 37∘C. Amplified telomerase products were fractionated in 10% non-denaturing PAGE (19:1, acrylamide:bis-acrylamide) in 1X TBE (100 mM Tris, 100 mM Boric acid, 2 mM EDTA disodium salt), transferred to nylon membranes, and incubated with anti-DIG-HRP conjugate antibody (Roche). Assays were developed using CPDstar (Roche).

PP (passage 6) extracts (∼1 μg) were also pre-incubated with 100 nM 17AAG for 30 min at 28°C before performing the TRAP assay to test if the inhibition of LHsp90 could disturb telomerase activity.

## Results

### Telomeres Are Distributed in Nuclear Clusters Throughout *L. amazonensis* Developmental Cycle and Show Differences in Length Among the Three Life Forms and During Continuous Passages

**FIGURE 1:**
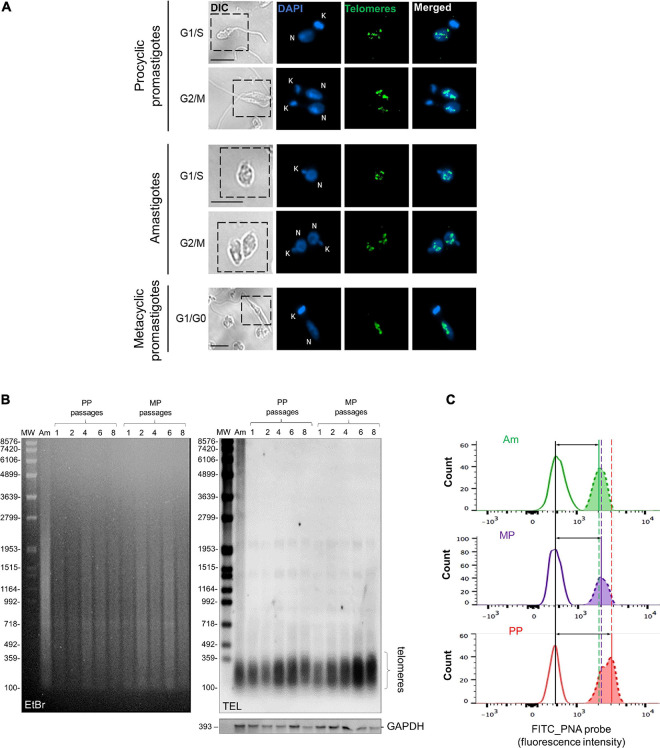
*L. amazonensis* telomeres distribution and size during development and continuous passages. **(A)** Distribution of *L. amazonensis* telomeres in three parasites developmental stage obtained using telomeric FISH. Telomeres were hybridized with a FITC-labeled telomeric oligoprobe (CCCTAA)_3_ (PANAGENE). DNA in the nucleus and kinetoplast were stained with Vectashield^®^ mounting medium DAPI (Vector Labs). The images were analyzed using a Nikon 80i fluorescence microscope and were superimposed using NIS elements software (v. Ar 3.10). Bar 2 μm. Telomeres are shown to form indistinct clusters and individual foci independent of the cell cycle phase and parasite life stages. **(B)** gDNA (1.0 μg) of lesion-derived Am, PP (passages 1–8), and MP (passages 1–8) were digested with *Afa*I (10 U). DNA fragments were separated on 0.8% ethidium bromide (EtBr)-stained agarose gel. Southern blotting hybridization was done with a DIG-labeled telomeric probe (5′-TTAGGG_3_-3′). As the loading control, a DIG-labeled probe that recognizes the *L. amazonensis* GAPDH gene was used. Chemiluminescent detection was performed using an anti-DIG specific antibody covalently coupled to alkaline phosphate (Roche) and CPD-Star (Roche). In addition, 1 kb plus DNA ladder and VII DIG-labeled were used as molecular weight markers (MW). **(C)** Flow-FISH analyses were performed with the three main parasite life forms using a PNA FITC-labeled (CCCTAA)_3_ telomeric probe (PANAGENE). Histograms represent the average fluorescence intensity of non-hybridized parasites (at the left) and the telomeric probe (at the right). The data were analyzed using FlowJo software v.7.6.5. Vertical lines mark the difference between peaks, and horizontal lines with arrows, represent telomere length differences in the populations analyzed. The amount of fluorescence among samples was calculate using MESF (Supplementary Table 1).

Using fluorescence *in situ* hybridization (FISH), we found that parasite telomeres are distributed in the nucleus of the three main *L. amazonensis* life forms, organized in multiple foci, as well as individual dots, or forming indistinct clusters. Telomeres distribution showed similar patterns throughout the cell cycle phases in Am and PP (Figure 1A). However, in MP, they were observed only at G1/G0 phase because this is a non-replicative form (Sacks and Perkins, 1984; Alvar et al., 2012) and, therefore, stays in a quiescent-like state.

Next, we used three different and complementary approaches to analyze telomere length during parasite development. The terminal restriction fragment (TRF) profiles of the three parasite life forms and from PP and MP maintained in continuous *in vitro* passages (1–8) were obtained using Southern blotting of gDNA hybridized with a telomeric probe using a standard protocol (Conte and Cano, 2005). The results shown in Figure 1B demonstrated that in lesion-derived Am, most of the telomere-containing fragments (Figure 1B, lane 1) range in size from ≤ 0.16 to 0.30 kb. Telomeres from PP with continuous passages (1–8) range in size from ≤ 0.16 to ≥ 0.38 kb (Figure 1B, lanes 2–6), whereas in MP from passages 1–8, telomeres range in size from ≤0.16 to 0.34 kb in length (Figure 1B, lanes 7–11; Supplementary Table 1). The results suggest that PP telomeres are slightly longer than Am and that parasite’s telomeres elongate after continuous *in vitro* passages. Telomere-associated sequences up to the first subtelomeric *Afa*I restriction site (Conte and Cano, 2005) appear as faint individual bands ≥ 1,0 kb (Figure 1B, lane 1–11). As the control, we hybridized the same DNA samples with a DIG-labeled fragment of *L. amazonensis* GAPDH (Figure 1B, bottom).

A qPCR assay was standardized to get a more precise result about the observed alterations in telomere length during parasite development (Aubert et al., 2012). It was possible to compare telomere amplification (T) with a single gene (S) amplification by qPCR to obtain the T/S ratio. Different *L. amazonensis* single gene candidates were tested: GAPDH, G6PDH, alpha-tubulin, and histone H2B (data not shown). The best parameters were obtained with GAPDH. The assay showed a confinable correlation of *R*^2^ = 0.99219, slope = −3.363 and amplification efficiency of 98% (Supplementary Figure 2). The qPCR results gave T/S ratios of, respectively, 1.64 and 0.54 for PP (passage 6) and Am (Supplementary Table 1). This result corroborates the TRF measurements shown in Supplementary Table 1, suggesting that PP telomeres are longer than Am.

Subsequently, we used a modified protocol of Flow-FISH assay (Baerlocher et al., 2006) to confirm the TRF measurements and qPCR results. Although all three methods are complementary, it is known that Flow-Fish is more precise and accurate than qPCR to measure telomere length in humans (Gutierrez-Rodrigues et al., 2014). Therefore, to ensure an accurate analysis of the data, human leukocytes were the internal control and processed together with parasite samples as described before (da Silva et al., 2017). Figure 1C shows FITC fluorescence histograms of the three *L. amazonensis* life forms hybridized with the PNA-FITC labeled telomeric probe. Each first peak in the graphs represents non-hybridized cells, whereas the second peaks represent the average fluorescence intensity emitted by the telomeric probe, which is proportional to telomere length. Therefore, the variation between peaks represents telomere length differences in the populations analyzed being, PP > MP > Am. The MESF values and the proportional change obtained for each sample are summarized in Supplementary Table 1.

In conclusion, it was possible to show that according to the average length of TRFs, the qPCR and Flow-FISH results (summarized in Supplementary Table 1), Am telomeres are shorter than PP and MP, strongly suggesting that telomere length is regulated during *L. amazonensis* development.

### Telomerase Activity Was Detected in All Main Life Forms of *L. amazonensis*

**FIGURE 2:**
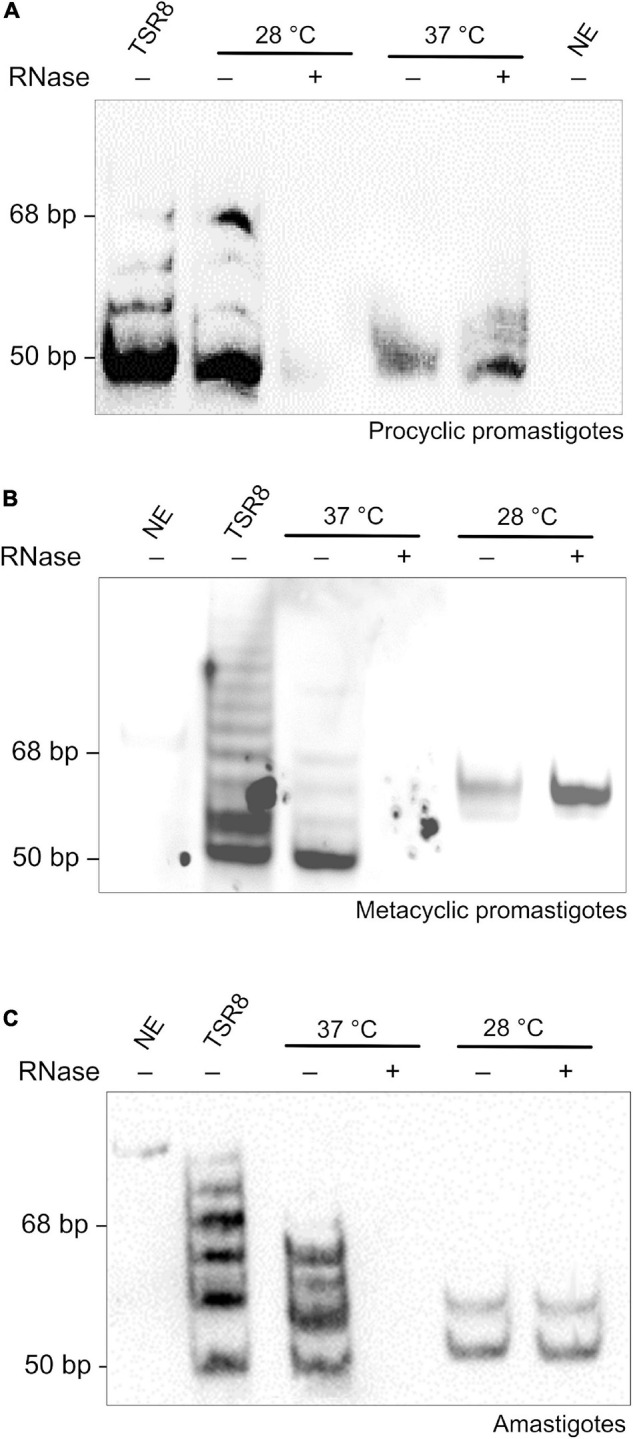
A temperature-dependent telomerase activity maintains telomeres in *L. amazonensis* spp. **(A–C)**. Telomerase activity was detected in semi-purified extracts using a modified one-tube Telomere Repeat Amplification Protocol (TRAP) assay (Cano et al., 1999; Giardini et al., 2011). Telomerase products were fractionated in a 10% non-denaturing PAGE in 1X TBE. Products were visualized using chemiluminescence. The amplification of a DIG-labeled TSR8 oligonucleotide was used as the 6-base increment ladder marker. **(A)** Telomerase activity detected in PP extracts. Lane 1, DIG-labeled TSR8. Lane 2, the reaction was done at 28∘C with the purified protein extract. Lane 3, the reaction was done at 28∘C with RNase A pre-treated extract. Lane 4, the reaction was done at 37∘C with the purified protein extract. Lane 5, the reaction was done at 37∘C with RNase A pre-treated extract. Lane 6, the reaction was done in the absence of extract (NE, no extract). **(B)** Telomerase activity detected in MP extracts. Lane 1, the reaction was done in the absence of extract (NE, no extract). Lane 2, DIG-labeled TSR8. Lane 3, the reaction was done at 37∘C with the purified protein extract. Lane 4, the reaction was done at 37∘C with RNase A pre-treated extract. Lane 5, the reaction was done at 28∘C with the purified protein extract. Lane 6, the reaction was done at 28∘C with RNase A pre-treated extract. **(C)** Telomerase activity detected in Am extracts. Lane 1, the reaction was done in the absence of extract (NE, no extract). Lane 2, DIG-labeled TSR8. Lane 3, the reaction was done at 37∘C with the purified protein extract. Lane 4, the reaction was done at 37∘C with RNase A pre-treated extract. Lane 5, the reaction was done at 28∘C with the purified protein extract. Lane 6, the reaction was done at 28∘C with RNase A pre-treated extract.

Subsequently, protein extracts of all three parasite life forms were tested for the presence of telomerase activity. Parasite extracts were obtained using an improved version of a previously standardized TRAP Assay protocol (Cano et al., 1999; Giardini et al., 2011). In this case, DEAE semi-purified protein extracts were tested for the presence of telomerase activity using the temperatures that parasite life forms live in their hosts (Wheeler et al., 2010). The results showed that PP telomerase was active at 28∘C but not at 37∘C (Figure 2A). In contrast, MP’s and Am’s telomerase was detected at 37∘C but not at 28∘C (Figures 2B,C). As controls, extracts were pre-treated with RNase A, which abolishes enzyme activity, and reactions were done in the absence of extract (NE, no extract) (Figures 2A–C). The bands in the lanes where the PP extracts were incubated at 37∘C (Figure 2A) and Am and MP extracts at 28∘C (Figures 2B,C) are not due to telomerase activity. The corresponding bands are probably artifacts or spurious PCR products (i.e., primer-dimers) (Kim and Wu, 1997; Krupp et al., 1997; Cano et al., 1999; Herbert et al., 2006; Giardini et al., 2011). TSR8 primer was used as an amplification marker since it produces a ladder of products with 6-base increments starting at 50 bp, the shortest band in the telomerase-positive extracts (Figures 2A–C). The results obtained show that telomerase activity can be detected in all *L. amazonensis* life forms. Also, for the first time, we present strong evidence that telomerase activity in *L. amazonensis* is probably life-stage dependent since in PP enzyme activity is detected at 28∘C, and in MP and Am, enzyme activity is detected at 37∘C.

### LHsp90 Can Be a Potential Telomerase Partner and Regulator of Telomere Length

To analyze if, similar to Hsp90 from model organisms (Holt et al., 1999; Toogun et al., 2008), LHsp90 could also be a telomerase partner, parasites were treated with increased concentrations of 17AAG, an analog of geldanamycin. 17AAG inhibits Hsp90 in model eukaryotes (Neckers and Neckers, 2002; Guo et al., 2005) and shows lethal effects on *L. amazonensis* life forms depending on the concentration used (Wiesgigl and Clos, 2001; Li et al., 2009; de Petersen et al., 2012; Santos et al., 2014).

**FIGURE 3:**
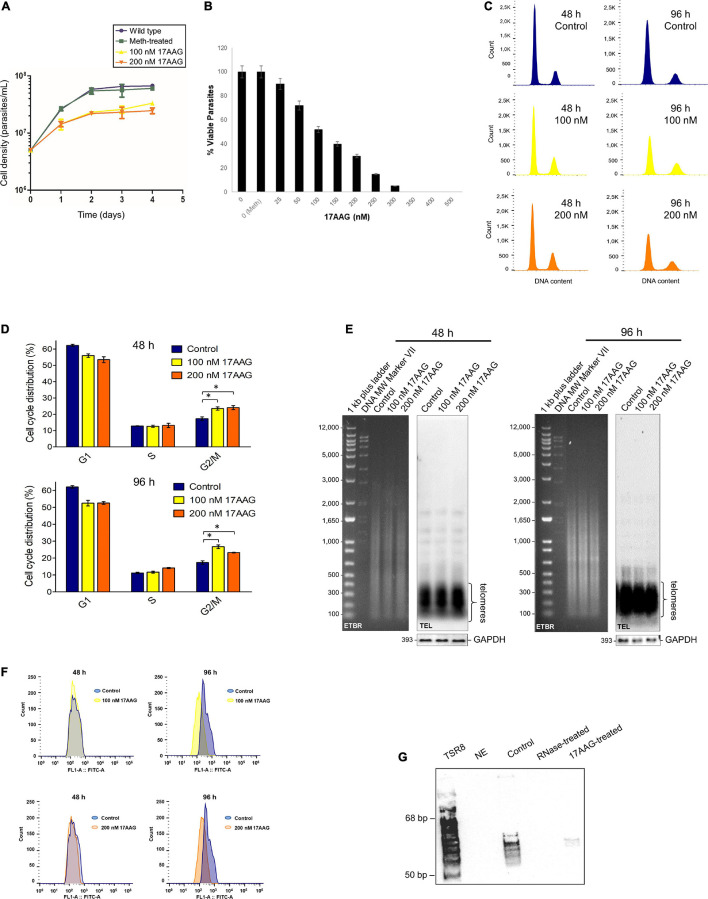
The inhibitor of HSP90, 17AAG, induces cell death, cell cycle arrest, telomere shortening, and telomerase inhibition in *L. amazonensis* PP. **(A)** Growth curves of PP non-treated (Wt and meth-treated parasites) and treated with 17AAG), maintained in exponential growth for 96 h and counted in the Neubauer chamber every 24 h. Wt and meth-treated parasites were used as the control. Treated parasites were cultivated in the presence of 100 and 200 nM 17AAG. **(B)** Cells’ viability test. *L. amazonensis* PP (∼3 × 10^6^ cells) in exponential growth were treated for 48 h at 28°C with increased concentrations of 17AAG. As a control, cells were grown in the absence (0) and the presence of methanol (0 Meth). The assay was done using PrestoBlue (Invitrogen) according to the manufacturer’s instructions. The percentage (%) of viable cells was estimated and plotted using Graphpad Prism 8. The error bars indicate the S.D. of the mean of triplicate samples. **(C)** The histograms represent cells non-treated and treated with 17AAG in each cell cycle phase based on a flow cytometer’s DNA content analysis of propidium iodide stained cells. The data were analyzed using FlowJo software v.7.6.5. **(D)** The graphs represent the percentage of PP (non-treated and treated with 17AAG) distributed in each cell cycle phase. As in **(C)** treated cells were incubated with 100 and 200 nM 17AAG for 48 and 96 h. Control represents cells grown in the presence of 90% methanol (meth-treated). **(E)** *Afa*I digested gDNA (1.0 μg) of meth-treated parasites and parasites treated for 48 and 96 h with 100 and 200 nM 17AAG. DNA fragments were separated on 0.8% EtBr (ethidium bromide)-stained agarose gels followed by Southern blotting hybridization with a DIG-labeled TEL probe (5′-TTAGGG_3_-3′). As the loading control, a DIG-labeled probe that recognizes the *L. amazonensis* GAPDH gene was used. Chemiluminescent detection was performed using an anti-DIG specific antibody (Roche) and CPD-Star (Roche). 1 kb plus DNA ladder (Invitrogen) and VII DIG-labeled Molecular weight (Roche) were used as molecular weight markers (MW). **(F)** Flow-FISH analyses were performed in triplicates using three different biological replicas. Parasite samples treated for 48 and 96 h with 100 and 200 nM 17AAG and meth-treated controls were hybridized using a PNA FITC-labeled (CCCTAA)_3_ telomeric probe (PANAGENE). Histograms represent the average fluorescence intensity emitted by non-hybridized parasites (control, blue) and by the telomeric probe (100 nM, yellow and 200 nM, orange). The data were analyzed using FlowJo software v.7.6.5. The amount of fluorescence among samples was calculate using MESF (Supplementary Table 3). **(G)** Telomerase activity was detected in DEAE-purified PP extracts using a modification of the one-tube TRAP assay (Cano et al., 1999; Giardini et al., 2011). Telomerase products were fractionated in a 10% non-denaturing PAGE in 1X TBE. Products were visualized using chemiluminescence. A DIG-labeled TSR8 oligonucleotide was used as the 6-base increment ladder marker. Lane 1, the reaction was done in the absence of extract (NE, no extract). Lane 2, the reaction was done at 28∘C with the telomerase positive protein extract. Lane 3, the reaction was done with the telomerase-positive protein extract pre-treated with RNase A. Lane 4, reaction done with the telomerase-positive extract pre-treated with 100 nM 17AAG.

The IC_50_ of 17AAG for *L. amazonensis* PP (passage 6) was estimated as ∼100 nM (Supplementary Figure 3). After that, parasites in exponential growth cultures were treated with 100 and 200 nM 17AAG for 48 and 96 h, followed by cell counting (Figure 3A), analyses of cell viability (Figure 3B) and the cell cycle (Figure 3C and Supplementary Table 2). The results showed a decrease in the number of cells after treating parasites with 100 and 200 nM 17AAG for 48 < 96 h, meaning that LHsp90 inhibition was time-dependent. As shown after DNA content analysis by flow cytometer, part of the cells treated for 48 and 96 h were arrested in G2/M (Figure 3C and Supplementary Table 2). The quantitative differences in DNA content in each cell cycle phase (G1, S, and G2/M) between treated, meth-treated, and wt parasites were obtained from the histograms (events × FL2-area) are shown in Figure 3D and summarized in Supplementary Table 2. It is worth informing that PP grown in the presence of 90% methanol (meth-treated) and wild-type cells (wt) showed identical DNA content profiles explaining why the experiments in Figures 3C–G were done with meth-treated parasites as the control.

We also checked if the inhibition of LHsp90 caused alteration in parasite telomere length. Southern blots using gDNA obtained from meth-treated, and PP treated for 48 and 96 h with 100 and 200 nM 17AAG were hybridized with a telomeric probe (TEL), and as the control, the same DNA samples were hybridized with an *L. amazonensis* DIG-labeled GAPDH probe. The results showed that LHsp90 inhibition induces minor alteration in PP telomere length (Figure 3E). However, Flow-FISH (Figure 3F) analysis showed that LHsp90 inhibition induced telomere shortening in *L. amazonensis* PP in a time-dependent manner. Furthermore, MESF analysis (Supplementary Table 3) showed a more accentuated decrease in telomere length after 96 h of treatment independent of drug concentration. Besides, 100 nM 17AAG abolished PP’s telomerase activity *in vitro* (Figure 3G), strongly suggesting that LHsp90 regulates parasite telomere length.

**FIGURE 4:**
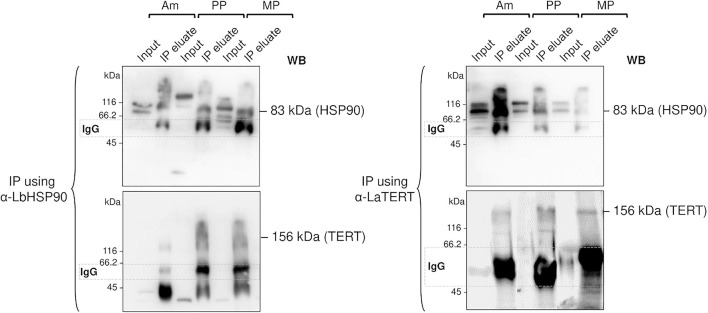
LHSP90 and the telomerase TERT component co-immunoprecipitated in the three *L. amazonensis* life forms. Co-immunoprecipitation (IP) assays were done using DynaBeads protein A, 20 μg of each telomerase positive extracts (input) obtained from Am, PP and MP, and specific polyclonal sera against LbHSP90 (left) or LaTERT (right). The total amount of IP eluates and 20 μg of each protein extracts (used as input) were fractionated in 10% SDS-PAGE and submitted to western blot analysis revealed with anti-LaTERT and anti-LbHSP90 sera. Goat anti-rabbit HRP-conjugate was used as the secondary antibody (Bio-Rad). The results were detected using enhanced chemiluminescence (ECL), according to the manufacturer’s instructions.

Therefore, to check if LHsp90 is part of the *L. amazonensis* telomerase complex, we carried out co-immunoprecipitation assays (Figure 4) using telomerase-positive extracts obtained from the three main parasite life forms. The results showed that LHsp90 and TERT co-immunoprecipitated in the three extracts, evidencing that both proteins are part of the same protein complex. The presence of co-migrating bands in the extracts (input) of all three parasite forms revealed with anti-LdHsp90 serum is noted. Post-translational modifications are commonly observed in Hsp90 from most organisms, including *Leishmania* spp. (Mollapour and Neckers, 2012; Hombach-Barrigah et al., 2019). Whether post-translational modifications are determinant for LHsp90/TERT interactions is still an open question. Curiously, TERT is apparently expressed in lower levels in all parasite forms, being detected only in highly concentrated extracts (∼300 μg/gel lane) (Supplementary Figure 4). On the other hand, LHsp90 can be detected in ten times less concentrated protein extracts (input lanes in Figure 4). This finding corroborates other studies that evidenced LHsp90 as a highly abundant protein in *Leishmania* spp. promastigotes, representing 2.8% of its total protein content (Brandau et al., 1995). It is worth mentioning that the intense non-specific bands presented below the hsp90 band in the western blot of the IP using anti-Lbhsp90 IgG, and below TERT in the western blot of the IP using anti-LaTERT, are probably IgGs that were detected by the HRP-conjugated anti-rabbit IgG secondary antibody since the primary antibodies used (α-LbHSP90 and α-LaTERT) are polyclonal.

Altogether, the results presented here strongly suggest that LHsp90 can be part of the telomerase ribonucleoprotein complex and acts as a potential regulator of *L. amazonensis* telomere length.

## Discussion

The present study used the three main parasite life forms (PP, MP, and Am) to investigate telomere length maintenance in *L. amazonensis*. We figured out that *L. amazonensis* telomeres are distributed in nuclear clusters in the three life forms regardless of the cell cycle phase (Figure 1A). Curiously, as revealed by qPCR, telomeres from Am are shorter than PP, and using Flow-FISH, we confirmed that Am telomeres are shorter than MP and PP (Supplementary Figures 1C, 2 and Supplementary Table 1). It was also observed by TRF analysis that telomeres from PP and MP maintained in continuous *in vitro* passages increased over time (Figure 1B). These differences in telomere length are not due to lack/inhibition of telomerase since enzyme activity was detected in the three life forms. However, the catalysis seems to be life-stage dependent (Figures 2A–C). It is worth reminding that MP is pre-adapted to face 37°C during PP-to-Am differentiation and that Am is fully adapted at 37°C, but not PP, nectomonads, and other promastigote forms (Inbar et al., 2017). Also, Genest and Borst (2007) had previously shown that telomeres from *Leishmania* PP elongate over time, and Bussotti et al. (2018) recently demonstrated that *Leishmania* spp. amplifies the subtelomeric/telomeric regions during adaptation in the host, using probably an unknown replication/recombination mechanism. Thus, unknown factors should be involved in parasite telomere length regulation. Here we speculate that the telomere length differences among parasite life forms and parasites in continuous *in vitro* passages may also be due to variations in telomerase holoenzyme biogenesis and composition during parasite development and growth. However, further assays are necessary to determine if the enzyme’s catalysis is dependent on the parasite life stage or the composition of the RNP complex, or both.

In model eukaryotes, besides the telomeric proteins, telomerase activity can be regulated by protein partners involved in the assembly/disassembly of the telomerase ribonucleoprotein complex (Smogorzewska and de Lange, 2004; Collins, 2006; Podlevsky and Chen, 2012; Viviescas et al., 2019; Nguyen and Wong, 2020). Some of these partners are conserved, and among them, there are molecular chaperones such as Hsp90 (Toogun et al., 2008) and chaperone-like proteins (Viviescas et al., 2019). It was previously demonstrated that human Hsp90 (hHsp90) remains associated with a functional telomerase complex. Hsp90 is responsible for the nuclear transport of TERT by importin alpha and enhances telomerase activity by helping the phosphorylation of TERT by the AKT protein kinase. Hsp90/AKT, in its turn, regulates TERT subcellular distribution (Haendeler et al., 2003; Keppler et al., 2006; Xi et al., 2013), avoiding the degradation of TERT by the ubiquitin-proteasome system (Viviescas et al., 2019). Besides, the reconstitution of an active human telomerase ribonucleoprotein complex is dependent on Hsp90 and its co-chaperone p23 (Toogun et al., 2008). The dissociation of p23 from the complex, in contrast, retains the TERT component in the cytoplasm (Akalin et al., 2001; Keppler et al., 2006). Early studies in budding yeast demonstrated that the overexpression of Hsp82 (the ortholog of Hsp90 in yeast) induced telomere shortening without affecting telomerase activity (Grandin and Charbonneau, 2001). Later on, Hsp82 was shown to help the association of telomerase with telomeres facilitating telomere elongation through its interaction with CDC13, an essential component of the telomere capping complex (Dezwaan et al., 2009). Thus, in budding yeast, Hsp82, besides telomerase, may affect other factors involved in telomere length maintenance (DeZwaan and Freeman, 2010). Notably, the pharmacological inhibition of Hsp90 by geldanamycin or one of its analogs (e.g., 17AAG) triggers telomere shortening and abolishes telomerase activity in both humans and yeast (Holt et al., 1999; Toogun et al., 2008), confirming the influence of the chaperone in important telomere functions (Holt et al., 1999; Forsythe et al., 2001).

Based on this knowledge, we hypothesize that the ortholog of Hsp90 in *L. amazonensis* (LHsp90) could be a telomerase partner responsible for regulating telomerase activity. Our findings argue in favor of a conserved role for LHsp90 at parasite telomeres since its inhibition induces parasite growth arrest, telomere shortening, and abolishes PP telomerase activity *in vitro* (Figures 3A–F). We also showed that LHsp90 and the telomerase TERT component co-immunoprecipitated in telomerase positive extracts obtained from the three main parasite life forms (Figure 4), evidencing that LHsp90 can be part of the telomerase complex. Moreover, the western blot analysis showed that LHsp90, like other Hsp90, probably suffers post-translational modifications (co-migrating bands in input lanes in Figure 4 and Supplementary Figure 4), which can be important to regulate its activity, and also its interaction with other client proteins, such as the TERT. As highly documented, post-translational modifications regulate Hsp90 functions in the different biological processes (Backe et al., 2020). However, further studies are needed to evidence if LHsp90 is part of the parasite telomerase ribonucleoprotein complex and affects other parasite telomeric proteins.

Like most protozoa parasites, *Leishmania* uses LHsp90 during stage transitions in their developmental cycles (Zilka et al., 2001). Since *Leishmania* lives and multiplies in different environments and temperatures, these stressful living conditions are probably detrimental to the interactions between protein partners and the assembly of specific protein complexes. Thus, we speculate that the composition of the telomerase ribonucleoprotein complex alters during parasite development, and LHsp90 could be an important player in the control of telomere length maintenance and parasite life span. Therefore, a detailed understanding of telomerase biogenesis and LHsp90 intersections with the telomeric machinery will greatly contribute to our knowledge of *Leishmania* spp. telomeres and how they might be subject to manipulation for therapeutic purposes. Telomeres and the proteins involved with telomere length maintenance in humans have been used as important targets for developing therapies against cancer and other telomere-related diseases (Martínez and Blasco, 2017; Fernandes S. G. et al., 2020). The *Leishmania* spp. telomeric machine, although share some conserved features with most eukaryotes, presents genus-specific characteristics, such as unique telomeric proteins (Morea et al., 2017; Fernandes et al., 2019, Fernandes C. A. H. et al., 2020), a singular telomerase RNA component (Vasconcelos et al., 2014), and amino acid substitutions in the TERT functional domains (Giardini et al., 2006), increasing the chance of finding a good and parasite-specific therapeutic target among these molecules.

## Data Availability Statement

The original contributions presented in the study are included in the article/Supplementary Material, further inquiries can be directed to the corresponding author/s.

## Author Contributions

BO, MES, and SP: investigation, methodology, validation, and writing—original draft preparation. MV: data curation, validation, and writing—original draft preparation. EM, CS, MS, and FG-R: investigation, methodology, and validation. MdS: investigation, methodology, validation, writing—reviewing, and editing. JB: resources, visualization, writing—reviewing, and editing. RC: resources, conceptualization, supervision, writing—reviewing, and editing. MINC: conceptualization, funding acquisition, project administration, supervision, writing—reviewing, and editing. All authors contributed to the article and approved the submitted version.

## Conflict of Interest

The authors declare that the research was conducted in the absence of any commercial or financial relationships that could be construed as a potential conflict of interest. The reviewer JA declared a shared affiliation with one of the authors, JB, to the handling editor at time of review.

## Publisher’s Note

All claims expressed in this article are solely those of the authors and do not necessarily represent those of their affiliated organizations, or those of the publisher, the editors and the reviewers. Any product that may be evaluated in this article, or claim that may be made by its manufacturer, is not guaranteed or endorsed by the publisher.
