# Corrigendum: Possible Involvement of Hsp90 in the Regulation of Telomere Length and Telomerase Activity During the *Leishmania Amazonensis* Developmental Cycle and Population Proliferation

**DOI:** 10.3389/fcell.2022.894949

**Published:** 2022-04-14

**Authors:** Beatriz C. D. de Oliveira, Mark E. Shiburah, Stephany C. Paiva, Marina R. Vieira, Edna Gicela O. Morea, Marcelo Santos da Silva, Cristiane de Santis Alves, Marcela Segatto, Fernanda Gutierrez-Rodrigues, Júlio C. Borges, Rodrigo T. Calado, Maria Isabel N. Cano

**Affiliations:** ^1^ Department of Chemical and Biological Sciences, Institute of Biosciences, São Paulo State University (UNESP), São Paulo, Brazil; ^2^ Faculdade Brasileira Multivix, Vitória, Brazil; ^3^ Hemocentro da Faculdade de Medicina de Ribeirão Preto, Universidade of São Paulo, São Paulo, Brazil; ^4^ São Carlos Institute of Chemistry, University of São Paulo, São Paulo, Brazil

**Keywords:** *Leishmania* life forms, continuous *in vitro* passages, telomeres maintenance, telomerase ribonucleoprotein complex, LHsp90

An author name was incorrectly spelled as **Stepany C. Paiva**. The correct spelling is **Stephany C. Paiva**.

The authors apologize for this error and state that this does not change the scientific conclusions of the article in any way. The original article has been updated.

