# Supplementary material for: Possible Involvement of Hsp90 in the Regulation of Telomere Length and Telomerase Activity During the Leishmania amazonensis Developmental Cycle and Population Proliferation
Source: Front Cell Dev Biol. 2021 Oct 28;9:713415. doi: 10.3389/fcell.2021.713415 (PMC8581162; doi:10.3389/fcell.2021.713415)
Supplement: Supplementary file 1 [file Data_Sheet_1.pdf]

Suppl. Figure 1

A

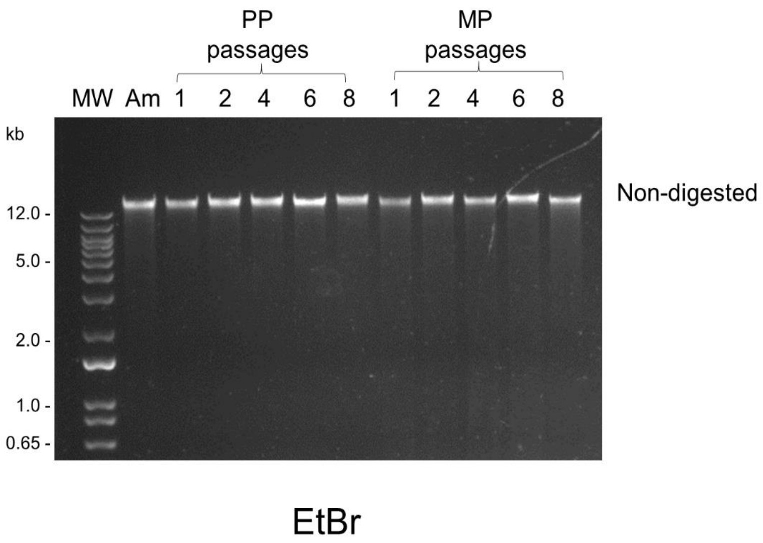

B

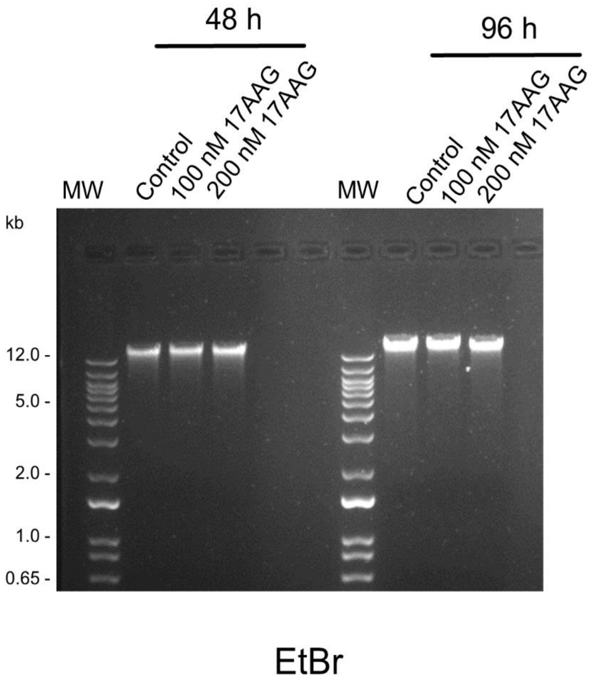

Suppl. Figure 1. The integrity of DNA samples used in the TRF analysis. A) Genomic DNA obtained from all three parasite life forms (A, amastigotes, PP procyclic promastigotes, MP, metacyclic promastigotes); PP and MP passages 1, 2, 4, 6, and 8. B) Genomic DNA obtained from PP non-treated (control) and treaded with 17AAG for 48 h and 96 h. The integrity of each non-digested DNA sample shown in A) and B) was confirmed by fractionation in 0.8% ethidium bromide (EtBr)-stained agarose gels. MW, 1kb plus DNA ladder (Invitrogen).

Suppl. Figure 2

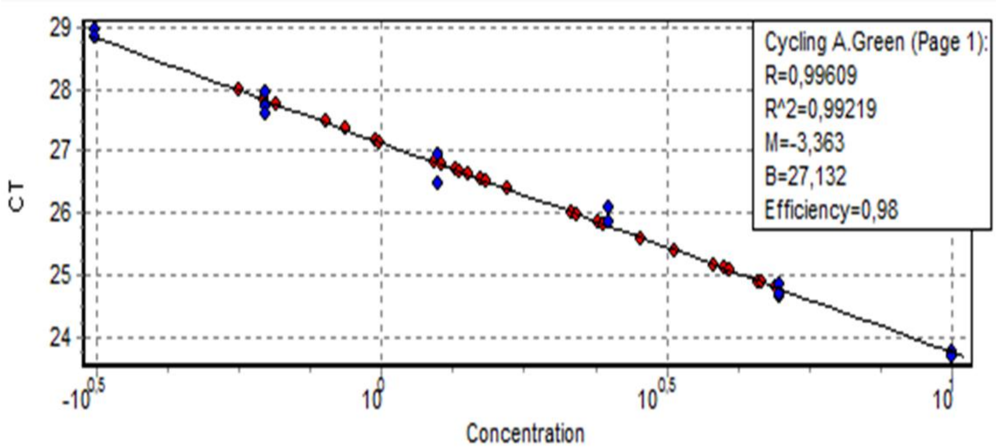

Suppl. Figure 2. qPCR telomere assay demonstrates that *L. amazonensis* promastigotes have longer telomeres compared to amastigotes. The telomere assay was performed on the Rotor-Gene® Q real-time instrument with the QIAGEN Rotor-Gene SYBR Green Kit. For the standard curve, gDNA from *Leishmania* promastigotes was diluted at 1:2 in six serial dilutions in the range of 10 ng-0.3125 ng/PCR in a 24 µl reaction volume. The PCR assay was repeated for 25 cycles. The assay was set up using a QIAgility® liquid handling instrument.

Suppl. Figure 3

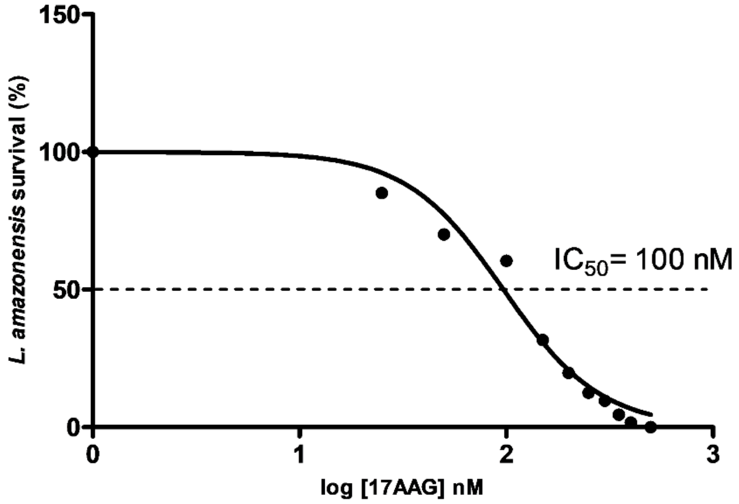

Suppl. Figure 3. The IC_{50} value of 17AAG for ***L. amazonensis*** **PP**. The IC_{50} value was determined from a dose-response inhibition fit using GraphPad Prism 8.0. Each value in the curve is the average of assays carried out in triplicates.

Suppl. Figure 4

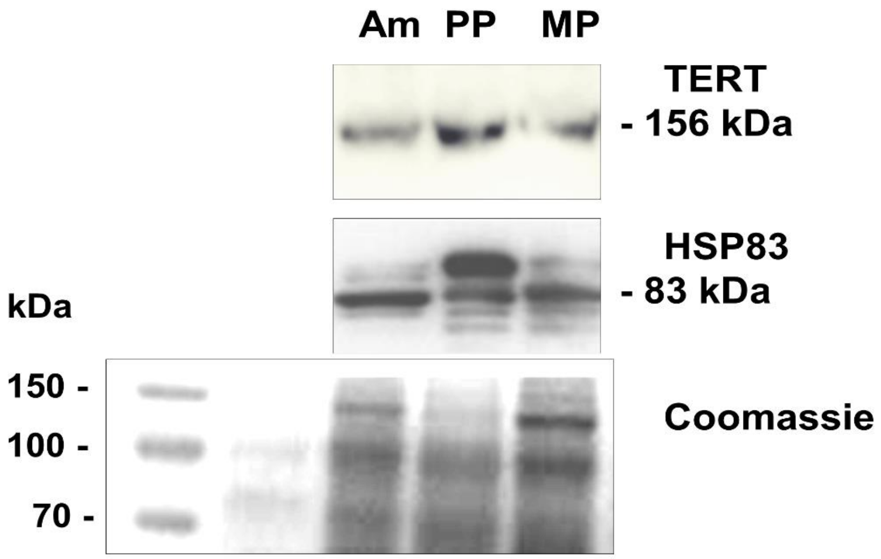

Suppl. Figure 4. Western blot analysis shows TERT and HSP83 in telomerase-positive extracts of the three parasite life forms. Approximately 300 µg of telomerase-positive extracts obtained from amastigotes (A), promastigotes (P), and metacyclics (M) were fractionated in 10% SDS-PAGE gels and transferred to nitrocellulose membranes (BioRad). Western blots were revealed with anti-LaTERT and anti-LdHSP83 polyclonal sera and developed using a goat anti-rabbit HRP-conjugated secondary antibody (Bio-Rad) and the enhanced chemiluminescence (ECL), according to the manufacturer's instructions (GE). Protein extracts fractionated in a Coomassie-stained 10% SDS-PAGE gel were used as the loading control.
