# Supplementary material for: Possible Involvement of Hsp90 in the Regulation of Telomere Length and Telomerase Activity During the Leishmania amazonensis Developmental Cycle and Population Proliferation
Source: Front Cell Dev Biol. 2021 Oct 28;9:713415. doi: 10.3389/fcell.2021.713415 (PMC8581162; doi:10.3389/fcell.2021.713415)
Supplement: Supplementary file 2 [file Data_Sheet_2.docx]

**Suppl. Table 1. Estimative of the telomere length in *L. amazonensis* life stages using**

**different approaches**

| **Method**  **Form** | **Southern Blotting**  **(TRF analysis*)** | **qPCR (T/S)** | **Flow-FISH**  **(MESF analysis)** | **Proportional change**  **(MESF analysis)** |
| --- | --- | --- | --- | --- |
| **PP** | > 0.16 - > 0.40 kb | 1.64 | 1,8155 + 1980 au | 1.3 |
| **MP** | > 0.14 - > 0.34 kb | ---- | 1,5061 + 1753 au | 1.1 |
| **Am** | > 0.12 - 0.30 kb | 0.54 | 1,3869 + 1,381au | 1.0 |

Kb: kilobases; au: arbitrary units

*TRF analysis considered the minimum and maximum size of the hybridized telomeric fragments

**Suppl. Table 2. Cell cycle analysis of *L. amazonensis* PP treated and non-treated with 17AAG. Data are presented as the Mean + SD**

**48h 96h**

| **Cell cycle phase** | **Meth-**  **Treated*** | **100 nM**  **17AAG** | **200 nM**  **17AAG** | **Meth-**  **Treated*** | **100 nM 17AAG** | **200 nM 17AAG** |
| --- | --- | --- | --- | --- | --- | --- |
| **G1** | 62.23 ±  1.159 | 55.93 ±  1.935** | 53.47±  3.166** | 62.10 ± 1.323 | 52.50 ± 2.905** | 52.63 ± 1.305** |
| **S** | 12.77 ±  0.251 | 12.63 ±  1.102 | 13.17 ±  2.230 | 11.20 ± 0.7211 | 11.67 ±  1.012 | 14.10 ± 0.6000** |
| **G2/M** | 13.17 ±  2.230 | 23.50 ± 1.709** | 24.07 ± 2.136** | 17.33 ± 1.823 | 26.73 ± 1.856** | 23.27 ± 0.3512** |

*PP grown in the presence of 90% methanol (drug diluent)

***P* values < 0.05 calculated using paired t-test and GraphPad (version 8.02)

**Suppl. Table 3. Flow-FISH (MESF analysis) of *L. amazonensis* PP treated**

**and non-treated with 17AAG for 48h and 96h**

|  | **MESF analysis** | **Proportional change**  **(MESF analysis)** |
| --- | --- | --- |
| **48h**  **Meth-treated*** | 2,482 ± 2,227 au | 1.0 |
| **100 mM** | 2,263 ± 1,980 au | 0.91 |
| **200 mM** | 2,206 au | 0.88 |
| **96h**  **Meth-treated*** | 2,649 au | 1.0 |
| **100 mM** | 1,887 ± 1,881 au | 0.71 |
| **200 mM** | 1,941 ± 1,772 au | 0.70 |

*PP grown in the presence of 90% methanol (drug diluent) used as control

au: arbitrary units
